# Cirrhotic cardiomyopathy

**Published:** 2014

**Authors:** VLM Grancea-Iancu

**Affiliations:** * Department of Internal Medicine and Rheumatology, “Dr. I. Cantacuzino” Hospital; “Carol Davila” University of Medicine and Pharmacy, Bucharest, Romania

**Keywords:** cirrhosis, cardiomyopathy, liver, arrhythmias, infarction

## Abstract

Cardiomyopathy is a chronic disease of the myocardium characterized by an abnormal dilatation and thinning of the left ventricular (LV), associated with the contractile dysfunction of the muscle and loss of pump capacity, resulting in the occurrence of arrhythmias and myocardial infarction. It has many causes and can occur in the liver pathology inclusively.

## Introduction

For about 50 years, the deterioration of liver function was associated with the cardiovascular system functioning through hyperdynamic circulation [**1**]. It has been shown that patients with liver cirrhosis show a cardiac and circulatory dysfunction mainly governed by the peripheral vasodilation [**2**,**3**]. The hyperdynamic syndrome, caused by the liver dysfunction and the portal hypertension associated with the splanchnic vasodilation may contribute to the myocardial disturbances in liver cirrhosis. Central hypovolemia in association with hypotension leads to the activation of baroreceptors and the volume receptors of the vasoconstrictor systems, this aggravating the hyperdynamic circulation and cardiac contraction. 

The results of the clinical and experimental studies have shown that the impaired myocardial contractility and electrophysiological abnormalities in cirrhosis define the notion of “cirrhotic cardiomyopathy” [**4**,**5**]. This term defines chronic cardiac dysfunction, characterized by a decreased contractile responsiveness to stress and impaired diastolic relaxation associated with electrophysiological abnormalities such as QT prolongation, in the absence of any heart disease [**6**]. Biochemical and physiological abnormalities of liver cirrhosis can affect heart rate, contractility, management and cardiac repolarization.

Cardiac consequences of the hyperdynamic syndrome are the following:

1. Systolic dysfunction - is a consequence of excessive release of nitric oxide (NO) and TNF that causes a reduced vascular reactivity to adrenaline and angiotensin II, respectively, altered beta-adrenergic signal. This hypothesis is supported by cardiovascular reflex tests in patients with cirrhosis, who are affected [**5**].

- contribute to the left ventricular hypertrophy (LVH), decreased cardiac output (CO) and heart failure.

 2. Diastolic dysfunction - is caused by the activation of the sympathetic nervous system (SNS) and the renin-angiotensin-aldosterone system (RAAS) by hypovolemia.

 - characterized by delayed filling of the left ventricle due to left ventricular hypertrophy (LVH), subendocardial edema and destruction of collagen structure. 

- contributes to fluid retention and the development of ascites.

The criteria for the positive diagnosis of cirrhotic cardiomyopathy target systolic dysfunction and diastolic dysfunction and the supportive criteria are summarized in **Table 1** [**6**]. The systolic function is directly proportional with the heart rate and cardiac output (CO). At rest, cardiac pressures are normal and CO is increased in patients with liver cirrhosis [**6**]. In some patients, it was found that exercise increases the pressure in the left ventricle (LV), ejection fraction and heart rate due to SNS tone. In contrast, the cardiac performance is reduced due to a lower cardiovascular reactivity [**7**]. The administration of vasoconstrictor substances (angiotensin II, terlipressin) increases peripheral vascular resistance and LV afterload. The exercise or the pharmacological point of view may reveal a latent left ventricular dysfunction in patients with cirrhosis, expressed by increased LV end-diastolic volume and decreased ejection fraction (EF). The increase in the value of atrial natriuretic peptide (ANP) in some cirrhotic patients can express an atrial distension associated with hypovolemia [**8**,**9**]. Ventricular mass is increased in some cirrhotic patients, associated with septal hypertrophy, which can be reflected by the level of troponin I, which may reveal this damage. This marker is increased in patients with cardiac ischemia but also in cirrhosis. BNP and pro-BNP are sensitive markers of myocardial alteration and are significantly increased in patients with compensated liver cirrhosis [**10**]. Levels of these peptides are correlated with the severity of cirrhosis, the degree of cardiac dysfunction and myocardial hypertrophy, and hyperdynamic circulation. Therefore, the determination of BNP and pro-BNP is useful in screening patients with cirrhotic cardiomyopathy.

Systolic dysfunction is the latency in patients with liver cirrhosis but is evidenced by exercise or pharmacologically. Reducing systolic function can cause complications in patients with liver cirrhosis [**6**].

Diastolic dysfunction is caused by a decreased LV compliance and relaxation, causing an abnormal LV filling. The pathophysiological background of the diastolic dysfunction is increased myocardial wall stiffness, consequently myocardial hypertrophy, fibrosis and subendothelial edema [**11**]. Sodium retention in patients with cirrhosis may lead to myocardial hypertrophy and diastolic dysfunction (Finucci et al.). Doppler echocardiography showed impaired ventricular relaxation, decreased E / A ratio and a decreased diastolic filling in patients with liver cirrhosis [**12**]. The renin-angiotensin-aldosterone system (RAAS) and ANP are associated with diastolic dysfunction in cirrhosis [**13**]. Thus, there is evidence that the diastolic function is impaired in patients with cirrhosis and the diastolic dysfunction indicators can provide prognostic information that may affect the ventricular filling during the procedure.

The prognosis of patients with cirrhotic cardiomyopathy is reserved due to undiagnosed cardiac dysfunction, which may increase the risk of death. Recent studies have shown that approximately 50% of the patients with liver cirrhosis performing signs of cardiac dysfunction before liver transplantation and 7-21% die by heart attack after transplantation [**11**]. In the advanced forms of liver cirrhosis characterized by vasodilation, hypovolemia and hypotension RAAS which increases DC, contributes to the stimulation of hydro-saline retention and development of hepato-renal syndrome, also with poor prognosis.

**Table 1:** Diagnostic criteria for cirrhotic cardiomyopathy

SYSTOLIC DYSFUNCTION	-increasing CO at exercise or pharmacological stimuli
	-EF < 55%
DIASTOLIC DYSFUNCTION	- E/A < 1
	- lengthening the deceleration time > 200msec
	- isovolumetric relaxation time > 80 msec
CRITERIA FOR SUPPORT	- electrophysiological abnormalities
	- abnormal chronotropic response
	- electromechanical dissociation
	- QT prolongation
	- LAH
	- increased myocardial mass
	- increasing pro-BNP and BNP
	- increased troponin I
Legend: CO- cardiac output, EF- ejection fraction, E / A - ratio initial diastolic filling / atrial filling, LAH - left atrial hypertrophy, BNP- B-type natriuretic protein	

## Treatment

The management in cirrhotic cardiomyopathy is not well structured. Diuretics increase the elimination of sodium and improve cardiac function. Beta-adrenergic blockers are commonly used in patients with liver cirrhosis with portal hypertension, because of the lowering and prevention of bleeding by the rupture of esophageal varices. Beta-blockers lower heart rate and reduce QT prolongation. However, there are no studies to support the benefits of these therapies in cirrhotic cardiomyopathy. Currently, liver transplantation is the only therapeutic solution for associated cirrhosis and cardiomyopathy [**14**].

## Conclusions

There is overwhelming evidence on the presence of cardiomyopathy in patients with cirrhosis. Further studies are needed to deepen the mechanisms involved in the development of cirrhotic cardiomyopathy and the completion of therapeutic means of this condition. 
